# Food consumption patterns and their micronutrient content in India: Evidence from the household consumption expenditure surveys, 2011–12 and 2023–24

**DOI:** 10.1038/s41430-026-01732-3

**Published:** 2026-04-07

**Authors:** Mudit Kapoor, Sankar Rajan, Gaurav Dhamija, Neha Sareen, Ambuj Roy, Archna Singh, Shamika Ravi

**Affiliations:** 1 Center for Research on the Economics of Climate, Food, Energy, and Environment (CECFEE), Economics and Planning Unit, Indian Statistical Institute, Delhi Center, New Delhi, 110016 India; 2 Independent Health Consultant, New Delhi, India; 3 Department of Liberal Arts, Indian Institute of Technology Hyderabad, Telangana, India; 4 Department of Cardiology, All India Institute of Medical Sciences, New Delhi, 110029 India; 5 Department of Biochemistry, All India Institute of Medical Sciences, New Delhi, 110029 India; 6 Member, Economic Advisory Council to the Prime Minister, Government of India, New Delhi, India

**Keywords:** Nutrition, Public health

## Abstract

**Background:**

India’s food consumption landscape has transformed over the past decade, with implications for nutrition security. This article quantifies changes in household expenditure, consumption patterns across major food groups, and dietary sources of selected micronutrients.

**Methods:**

Data from nationally representative Household Consumption Expenditure Surveys (HCES) 2011–12 and 2023–24 were analysed. Food quantities were standardised to adult female equivalents, and micronutrient intake was derived by linking food acquisition data to Indian Food Composition Tables. Models were fitted using a generalised additive mixed model with random effects (bam function, *mgcv* package in R).

**Results:**

Monthly per capita expenditure has increased across rural and urban areas, while share of food in household expenditure has declined, signalling economic diversification. Within food budgets, share of cereals has fallen sharply, particularly among poorest quintiles, consistent with expansion of food security programmes providing subsidised cereals. Meanwhile, probability and quantity of consumption of nutrient-dense foods- dairy, fruits, and flesh products, has risen across income groups, with larger gains among poorest households. Seasonal and regional disparities in perishable food consumption persist but have narrowed. Despite greater dietary diversity, micronutrient intake remains suboptimal. Estimated daily intake of Iron, Zinc, Calcium, and B Vitamins fell below Estimated Average Requirements for non-lactating adult women, with median inadequacy exceeding 75% for several micronutrients.

**Conclusion:**

India’s dietary transition shows progress but persistent gaps. HCES-based estimates provide valuable insights, underscoring need for integrated strategies aligning food policy, social protection, and nutrition-sensitive interventions to improve equitable access to nutrient-dense foods and reduce micronutrient inadequacy.

## Introduction

Reliable dietary data remain scarce in many low and middle-income countries, including India, where nutrition policies are often formulated without robust, nationally representative evidence on actual food consumption. This gap limits the effectiveness of interventions aimed at improving dietary quality and addressing malnutrition. In this context, the Household Consumption Expenditure Survey (HCES), conducted periodically by the National Sample Survey Office (NSSO) under the Ministry of Statistics and Programme Implementation (MoSPI), Government of India [1], is an important resource for monitoring household food acquisition and expenditure. With large sample sizes and comprehensive coverage across states and socioeconomic strata, HCES provides detailed data that can be used to assess population-level patterns in food access. Although HCES is not designed to capture individual dietary intake, standardised methods—such as adult female equivalent (AFE) adjustments—help address differences in household demographic composition and support more meaningful nutritional analysis [2, 3].

Empirical measurement of nutrition outcomes typically relies on more direct and resource-intensive approaches. Detailed 24-h dietary recall surveys and biomarker-based assessments remain the gold standard for estimating individual-level dietary adequacy and nutritional status, but they are costly, logistically demanding, and infrequent, particularly in large countries such as India. Against this backdrop, HCES complements individual dietary surveys by enabling analysis of population-level dietary transitions, including shifts in consumption probability and quantities, seasonal variations, and inequalities by socioeconomic status and location. It also offers insight into changes within the food system that are relevant for nutrition policy, including the diversification of diets away from cereals. These data are not substitutes for individual-based assessments, but they provide the scale and periodicity needed to track whether the food environment is evolving in a direction that supports nutrition security.

Diet is a significant determinant of health, and poor dietary patterns are among the leading contributors to the avoidable disease burden [4–6]. In India, recent estimates suggest that unhealthy diets account for over half of the nation’s disease burden [7], with strong links to non-communicable diseases such as type 2 diabetes, cardiovascular disease, and hypertension [8]. Despite improvements in food availability and economic growth, micronutrient deficiencies persist in India, particularly among vulnerable groups. Efforts to improve nutritional outcomes must go beyond meeting basic nutrient requirements. They should also prioritise enhancing dietary diversity, particularly among economically disadvantaged populations who often face systemic barriers to accessing a variety of nutrient-rich foods. Without deliberate strategies to expand food choices and improve affordability, interventions risk reinforcing existing inequities rather than resolving them. In India, micronutrient deficiencies remain widespread, and understanding actual food consumption patterns is essential for designing effective, context-sensitive nutrition strategies. These must extend beyond the health sector to include agriculture policy, food logistics, social safety nets, and market access.

This article synthesises HCES data from 2011–12 and 2023–24 to quantify changes in household food expenditure, the probability and quantity of consumption across major food groups, and the dietary sources of selected micronutrients. Our objective is to characterise population-level dietary transitions and inequities, rather than to estimate individual intake or clinical nutritional status. The findings provide insight into how India’s food consumption patterns are evolving, where progress has occurred, and which gaps remain critical for food and nutrition policy action.

## Materials and methods

### Data sources

**Fig. 1:**
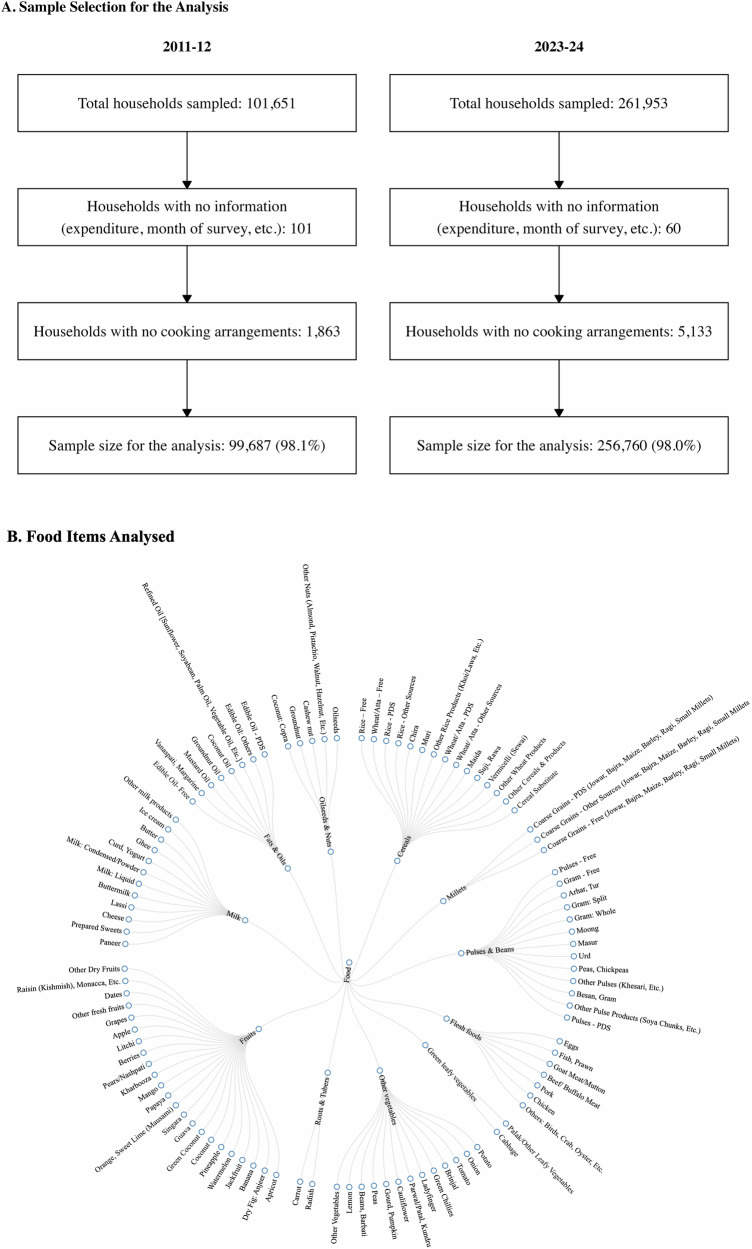
Sample Selection and the Food Items Analysed. **A** Schematic representation of households included in the analytical sample. **B** Detailed list of food items.

This review draws on unit-level data from two nationally representative surveys conducted by the NSSO: the 68th Round 2011–12 [9] and the HCES 2023–24 [10]. Both surveys employed a stratified two-stage sampling design covering all states and union territories, except for a few remote villages in the Andaman and Nicobar Islands. The 2011–12 round surveyed 101,651 households, while the 2023–24 round covered 261,953 households. After excluding households with missing data on monthly per capita expenditure (MPCE) or cooking arrangements, the final analytical samples comprised 99,687 and 256,760 households, respectively. See Fig. 1A for the sample selection for the analysis.

HCES collects detailed information on household demographics, food and non-food expenditures, and consumption quantities for over 175 food items (see Fig. 1B). Food items are grouped into 11 broad categories, with sub-item granularity (e.g., rice, wheat, coarse grains) enabling regional dietary profiling. Mixed recall periods were used: a 30-day recall for cereals and pulses, and a 7-day recall for all other food items. Notably, the 2023–24 survey used staggered household visits and computer-assisted personal interviewing (CAPI) to enhance data quality.

### Outcome and independent variables

The primary outcomes with respect to food items were: (i) probability of consumption and (ii) quantity consumed (kg per AFE over 30 days) for six food groups: cereals, pulses, vegetables, dairy (milk & milk products), fresh fruits, and flesh products (eggs, fish, meat). Our analysis relies on household-level food acquisition data from the HCES and implicitly assumes consumption within the recall period. Information on food wastage and storage is not observed in the data.

Nutrient acquisition was estimated by linking food quantities to composition values from the Indian Food Composition Tables (IFCT, 2017) [11, 12] supplemented by UK Food Composition tables and the USDA databases [13–16]. We focused on micronutrients of public health relevance in India: iron, zinc, calcium, folate, thiamine, riboflavin, niacin, and vitamin B6. Because nutrient composition values pertain to raw foods and no adjustments were made for cooking or processing losses, our estimates likely overstate true intake. Household size was standardised using AFE methodology to approximate individual-level intake [2]. AFE methodology provides a standardised approach for comparing households (See Supplementary Section 1 for additional details). However, it may systematically over- or under-estimate nutrient inadequacy for certain population groups, such as adolescents, the elderly, and men, whose nutritional requirements and intake distributions are not well represented by a female-centered adjustment. Seven-day recall data were scaled to 30-day equivalents. MPCE quintiles were constructed separately for each state-sector combination. Additional covariates included sector (rural/urban), state, and survey month.

### Statistical methods

We modelled the food intake in two steps. In the first step, we estimate the probability of food intake using a penalised logistic regression model. For the second step, we divided the total household consumption of the food item by the number of household members, expressed as AFE. Conditional on the positive amounts consumed by household members, the total food consumed in terms of AFE was modelled using two continuous distributions: the log-normal and the Gamma model with a log link.

The first and second steps were modelled using a random effects structure.

In particular, for the first step, we constructed a binary variable equal to 0 if the household did not consume the food item and one if it did. The probability of consuming each food item was estimated using a generalised additive mixed model (GAM) with the ‘bam‘ function from the ‘mgcv‘ package in R [17–19]. Random-effect smooths were included for the NSS region, rural/urban sector, economic class, and their pairwise interactions. For perishable items with substantial seasonal variation (e.g., fruits and vegetables), seasonal effects and their interactions were also included. NSS regions are defined within states for large states, while smaller states and Union Territories form a single NSS region. We construct the NSS region identifier as a state-specific code (state × NSS region), ensuring that regions are globally unique. Random effects at the NSS-region level therefore capture within-state regional heterogeneity for large states and reduce to state-level effects for smaller states and UTs. We also allow for sector-specific random effects and region-sector interactions, permitting sectoral differences to vary across NSS regions.

Models were fitted with a quasi-binomial family with a logit link, penalised likelihood estimation (fREML), and an increased penalty parameter (γ = 1.4) to limit overfitting. Computational efficiency was achieved through the use of the discrete=TRUE option, which is suitable for large samples.

For the second, households with positive consumption of food items were modelled using two alternative specifications (i): Gamma regression with log link, and (ii) Log-normal regression (where a Gaussian model was applied to log-transformed intake).

In both models, we used the random-effects structure. The final model was selected based on the Akaike Information Criterion (AIC), where the model with the lower AIC value was chosen. The random effects selected were the same as those in the probability model, and the models were estimated using a generalised additive mixed model (GAM) with the ‘bam‘ function from the ‘mgcv‘ package in R [17–19].

Next, we use the estimates from the probability model and the food intake model to construct the product of the predicted probability of food intake and the expected food intake in terms of the AFE. This was defined as the unconditional expected intake of the food item in terms of AFE. Along with the mean intake, we also report the corresponding 95% uncertainty interval.

All analyses adhered to the ethical standards for the use of secondary data. The datasets are publicly available via the Government of India’s microdata portal for HCES 2023–24 and NSS 68th Round for 2011–12 [20]. The analytical codes required for reproducibility will be made available upon request.

## Results

The results show a changing pattern in household expenditure. Between 2011–12 and 2023–24, we documented a rise in the average household expenditure in rural areas from Indian Rupees (INR) 7531 to INR 20,689 and in urban areas from INR 11,950 to INR 29,510. During the same period, the share of household expenditure devoted to food declined markedly—from 53.4% to 47.8% in rural areas and from 43.9% to 40.8% in urban areas (see Table 1). Concurrently, spending on non-food items such as consumables and durable goods increased. Notably, the share of expenditure on cereals halved over the decade, dropping from 11.24% to 5.13% in rural areas and from 7.37% to 4.04% in urban areas.

**Table 1:** Distribution of Household Expenditure Across Consumption Items, 2011–12 & 2023–24.

	Rural		Urban	
	2011–12	2023–24	2011–12	2023–24
Household Expenditure (Indian Rupees)	₹7531	₹20,689	₹11,950	₹29,510
Proportion of Household Expenditure Spent on Items				
Consumables	34.7%	39.1%	44.0%	46.4%
Pan	0.47%	0.52%	0.26%	0.26%
Tobacco	1.43%	1.50%	0.75%	0.91%
Intoxicants	1.17%	1.73%	0.61%	1.16%
Fuel And Light	7.82%	5.92%	6.91%	5.70%
Education	3.58%	3.47%	6.91%	6.25%
Medical (Hospitalisation)	2.23%	2.28%	2.10%	2.06%
Medical (Non-Hospitalisation)	4.42%	4.34%	3.61%	3.81%
Entertainment	0.93%	0.93%	1.52%	1.70%
Toilet Articles	2.05%	2.84%	2.11%	3.07%
Other Household Consumables	1.85%	2.25%	1.76%	2.08%
Consumer Services Excl. Conveyance	4.01%	5.20%	5.37%	5.61%
Conveyance	4.24%	7.50%	6.45%	8.45%
Rent	0.29%	0.39%	4.89%	5.02%
Consumer Taxes & Cesses	0.22%	0.19%	0.78%	0.31%
Durables	11.9%	13.1%	12.1%	12.8%
Clothing	5.81%	5.28%	5.27%	4.59%
Bedding Etc.	0.35%	0.42%	0.31%	0.35%
Footwear	1.07%	1.01%	1.05%	0.90%
Minor Durable-Type Goods / Sports Goods	0.32%	0.12%	0.29%	0.12%
Furniture & Fixtures	0.18%	0.42%	0.19%	0.38%
Goods For Recreation	0.15%	0.14%	0.21%	0.19%
Crockery & Utensils	0.22%	0.31%	0.15%	0.28%
Cooking & Other Household Appliances	0.20%	0.70%	0.39%	0.82%
Transport Equipment	1.31%	1.28%	1.96%	1.82%
Medical Equipment	0.00%	0.02%	0.00%	0.05%
Personal Goods	0.25%	1.07%	0.42%	1.22%
Residential Building, Land & Other Durables	0.85%	1.15%	0.68%	0.80%
Jewellery & Ornaments	1.22%	1.20%	1.16%	1.29%
Food	53.4%	47.8%	43.9%	40.8%
Cereals	11.24%	5.13%	7.37%	4.04%
Cereal Substitute	0.06%	0.02%	0.05%	0.02%
Pulses & Products	2.96%	2.09%	2.21%	1.51%
Milk & Milk Products	8.46%	8.89%	7.59%	7.84%
Salt & Sugar	1.91%	0.93%	1.28%	0.63%
Edible Oil	3.79%	2.84%	2.90%	1.95%
Egg, Fish & Meat	4.64%	4.88%	3.88%	3.70%
Vegetables	6.55%	6.04%	4.90%	4.32%
Fruits (Fresh)	2.21%	2.63%	2.61%	2.63%
Fruits (Dry)	0.59%	1.21%	0.81%	1.30%
Spices	3.37%	3.24%	2.50%	2.38%
Beverages	2.12%	2.39%	2.32%	2.65%
Served Processed Food	3.35%	4.23%	3.14%	4.53%
Packaged Processed Food	2.15%	3.27%	2.32%	3.29%

Consumption patterns across food groups indicate increasing diversification (see Tables 2A and 2B). Coverage of fresh fruit consumption among rural households rose from 68% (the 2.5% lower and the 97.5% upper bounds were 68% and 69%, respectively) to 93% [95% Uncertainty Interval (UI): 93–93], while for the poorest quintile it increased from 48% [95% UI: 47–50] to 86% [95% UI: 85–86]. Average monthly intake rose from 1.69 kg [95% UI: 1.65–1.73] to 2.35 [95% UI: 2.32–2.38] kg per AFE over 30 days, and the richest-to-poorest intake ratio narrowed from 4.05 (ratio of 2.96 kg for the top 20th percentile to 0.73 kg for the bottom 20th percentile) to 2.6 (ratio of 3.54 kg to 1.34 kg). Dairy products (Milk & Milk products) reached 95% [95% UI: 95–95] coverage in rural areas, with the poorest quintile gaining 1.46 kg per AFE over 30 days and reduced disparities.

**Table 2:** Proportion of Food Consumed and Quantity of Food Consumed (in terms of Adult Female Equivalent), 2011–12 & 2023–24.

A. Proportion of Households Consuming, 2011–12 & 2023–24				
	Proportion of Households Consuming (with 95% Uncertainty Interval)			
	Rural		Urban	
	2011–12	2023–24	2011–12	2023–24
**Fresh Fruits**				
Overall	68 (68–69)	93 (93–93)	84 (84–85)	96 (96–97)
Bottom 20%	48 (47–50)	86 (85–86)	69 (67–71)	92 (91–92)
Top 20%	85 (84–86)	97 (97–97)	95 (94–96)	99 (98–99)
**Milk & Milk Products**				
Overall	84 (84–85)	95 (95–95)	95 (94–95)	98 (98–98)
Bottom 20%	69 (68–70)	90 (89–90)	87 (86–88)	96 (96–97)
Top 20%	94 (94–95)	98 (98–98)	99 (98–99)	99 (99–99)
**Flesh products (Eggs, Fish & Meat)**				
Overall	54 (54–55)	75 (74–75)	59 (58–60)	73 (73–74)
Bottom 20%	45 (44–47)	72 (71–73)	57 (55–59)	76 (74–77)
Top 20%	59 (58–61)	75 (74–76)	55 (53–57)	69 (68–71)
**Vegetables**				
Overall	100 (100–100)	100 (100–100)	100 (100–100)	100 (100–100)
Bottom 20%	100 (100–100)	100 (100–100)	100 (100–100)	100 (100–100)
Top 20%	100 (100–100)	100 (100–100)	99 (99–100)	100 (99–100)
**Pulses & Pulse products**				
Overall	99 (99–100)	100 (100–100)	100 (100–100)	100 (100–100)
Bottom 20%	99 (99–99)	100 (100–100)	100 (99–100)	100 (100–100)
Top 20%	100 (99–100)	100 (100–100)	99 (99–100)	100 (100–100)
**Cereals**				
Overall	100 (100–100)	100 (100–100)	100 (100–100)	99 (99–99)
Bottom 20%	100 (100–100)	100 (100–100)	100 (100–100)	100 (99–100)
Top 20%	100 (100–100)	100 (100–100)	100 (100–100)	99 (99–99)
**Fat & Oils**				
Overall	100 (99–100)	100 (100–100)	100 (100–100)	100 (100–100)
Bottom 20%	100 (100–100)	100 (100–100)	100 (100–100)	100 (100–100)
Top 20%	99 (99–100)	100 (100–100)	99 (99–99)	100 (100–100)
**Oilseeds**				
Overall	24 (24–25)	72 (72–73)	39 (38–40)	83 (82–84)
Bottom 20%	17 (15–18)	62 (61–63)	29 (27–31)	75 (73–77)
Top 20%	33 (32–35)	81 (80–81)	54 (52–56)	89 (88–90)

B: Quantity of Food Consumed (in terms of Adult Female Equivalent), 2011–12 & 2023–24				
	**Quantity (kg) consumed in a month (AFE) (with 95% Uncertainty Interval)**			
	**Rural**		**Urban**	
	**2011–12**	**2023–24**	**2011–12**	**2023–24**
**Fresh Fruits**				
Overall	1.69 (1.65–1.73)	2.35 (2.32–2.38)	2.62 (2.55–2.68)	3.36 (3.3–3.42)
Bottom 20%	0.73 (0.7–0.78)	1.34 (1.32–1.37)	1.16 (1.1–1.22)	1.98 (1.92–2.04)
Top 20%	2.96 (2.87–3.05)	3.54 (3.48–3.6)	4.68 (4.52–4.83)	5.11 (4.97–5.24)
**Milk & Milk Products**				
Overall	4.96 (4.85–5.06)	5.67 (5.58–5.75)	6.91 (6.74–7.06)	6.83 (6.71–6.95)
Bottom 20%	2.22 (2.14–2.32)	3.68 (3.6–3.78)	3.14 (3.02–3.26)	4.43 (4.29–4.58)
Top 20%	8.28 (8.04–8.55)	7.88 (7.73–8.06)	11.91 (11.51–12.34)	9.68 (9.44–9.92)
**Flesh products (Eggs, Fish & Meat)**				
Overall	0.59 (0.58–0.61)	0.87 (0.86–0.89)	0.74 (0.72–0.76)	1.01 (0.98–1.04)
Bottom 20%	0.31 (0.3–0.33)	0.62 (0.6–0.63)	0.43 (0.41–0.45)	0.79 (0.76–0.82)
Top 20%	0.92 (0.89–0.95)	1.13 (1.11–1.16)	1.04 (0.99–1.1)	1.19 (1.14–1.25)
**Vegetables**				
Overall	6.51 (6.46–6.56)	6.85 (6.81–6.89)	6.64 (6.56–6.72)	6.78 (6.72–6.83)
Bottom 20%	4.99 (4.91–5.07)	5.44 (5.4–5.48)	4.92 (4.83–5.01)	5.42 (5.34–5.5)
Top 20%	8.16 (8.07–8.26)	8.42 (8.35–8.49)	8.46 (8.31–8.59)	8.3 (8.2–8.41)
**Pulses & Pulse products**				
Overall	0.75 (0.74–0.75)	0.7 (0.69–0.7)	0.87 (0.86–0.88)	0.76 (0.75–0.76)
Bottom 20%	0.57 (0.57–0.58)	0.56 (0.55–0.56)	0.67 (0.66–0.68)	0.62 (0.61–0.63)
Top 20%	0.96 (0.95–0.97)	0.85 (0.85–0.86)	1.09 (1.08–1.11)	0.9 (0.88–0.91)
**Cereals**				
Overall	10.75 (10.71–10.79)	8.81 (8.78–8.84)	8.86 (8.81–8.92)	7.56 (7.52–7.6)
Bottom 20%	9.97 (9.9–10.04)	8.23 (8.19–8.28)	8.66 (8.57–8.74)	7.41 (7.35–7.47)
Top 20%	11.31 (11.23–11.38)	9.25 (9.19–9.31)	8.7 (8.61–8.79)	7.5 (7.43–7.57)
**Fat & Oils**				
Overall	0.64 (0.63–0.64)	0.83 (0.82–0.83)	0.82 (0.81–0.83)	0.89 (0.89–0.9)
Bottom 20%	0.47 (0.47–0.48)	0.69 (0.69–0.7)	0.62 (0.61–0.64)	0.76 (0.75–0.77)
Top 20%	0.81 (0.8–0.82)	0.97 (0.96–0.97)	1.01 (1–1.03)	1.02 (1.01–1.04)
**Oilseeds**				
Overall	0.07 (0.06–0.07)	0.19 (0.18–0.19)	0.1 (0.1–0.11)	0.26 (0.25–0.26)
Bottom 20%	0.03 (0.03–0.04)	0.12 (0.11–0.12)	0.06 (0.06–0.07)	0.17 (0.16–0.18)
Top 20%	0.11 (0.1–0.12)	0.27 (0.26–0.27)	0.17 (0.16–0.18)	0.36 (0.35–0.38)

Note: 95% Uncertainty intervals are reported in parenthesis.

The share of households with any consumption of flesh products (Eggs, Fish, & Meat) increased by 27 percentage points among rural low-income households, with inequality (measured as the ratio of the highest to the lowest value) in quantity consumption (standardised by AFE) declining from a ratio of 3.0 to 1.8. Vegetable consumption showed modest gains with relatively stable inequality. Cereal consumption declined across all socioeconomic classes, while pulses maintained near-universal coverage and stable intake levels. These shifts were most pronounced among lower-income groups. Compared to 2011–12, the 2023–24 data revealed a marked reduction in inequity across key food groups—for instance, the ratio of dairy products consumption (standardised by AFE) of the top 20% and bottom 20% quintiles narrowed from 3.7 to 2.1 (a reduction by over 43%).

Approximately 30 percent of India’s population identifies as lacto-vegetarian [21], reflecting a structurally important dietary segmentation. This segmentation has significant implications for nutrient intake patterns, particularly with respect to animal-source foods. While the HCES does not collect self-reported vegetarian status, we partially address this heterogeneity by reporting the share of households with zero or positive consumption of flesh products (eggs, fish, and meat). Thus, changes in flesh product consumption should be interpreted as reflecting both shifts in participation and changes among consuming households.

Against this background, our analysis of dairy products (milk and milk products) and flesh products (eggs, fish, and meat) consumption reveals two distinct patterns, particularly among households in the Bottom 20% of the consumption distribution. For example, in rural areas, the proportion of Bottom 20% households consuming dairy products increased from 69% [95% UI: 68–70] in 2011–12 to 90% [95% UI: 89–90] in 2023–24, while average monthly consumption rose from 2.22 [95% UI: 2.14–2.32] kg to 3.68 [95% UI: 3.60–3.78] kg per AFE over 30 days. In urban areas, the corresponding share increased from 87% [95% UI: 86–88] to 96% [95% UI: 96–97], and average monthly consumption rose from 3.14 [95% UI: 3.02–3.26] kg to 4.43 [95% UI: 4.29–4.58] kg per AFE over 30 days.

Over the same period, the proportion of bottom-20-percent households consuming flesh products increased from 45% [95% UI: 44–47] to 72% [95% UI: 71–73] in rural areas, with average monthly consumption rising from 0.31 [95% UI: 0.30–0.33] kg to 0.62 [95% UI: 0.60–0.63] kg per AFE over 30 days. In urban areas, the share increased from 57% [95% UI: 55–59] to 76% [95% UI: 74–77], while consumption rose from 0.43 [95% UI: 0.41–0.45] kg to 0.79 [95% UI: 0.76–0.82] kg per AFE over 30 days.

**Fig. 2:**
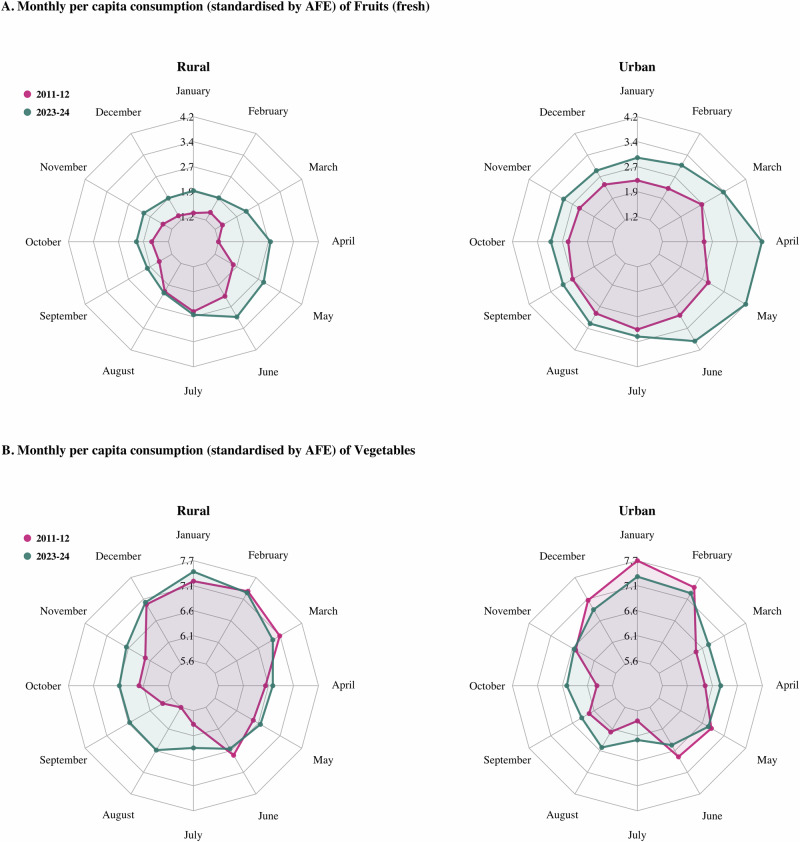
Seasonality in Consumption (in terms of Adult Female Equivalent), 2011–12 & 2023–24. **A** Fresh fruits. **B** Vegetables.

Seasonal variation remains a determinant of perishable food intake (Fig. 2 and Supplementary Table 2). Urban fresh fruit consumption peaked at 4.15 [95% UI: 4.00–4.31] kg per AFE in May and declined to 2.88 [95% UI: 2.75–3.02] kg in December, while rural peaks reached 3.02 [95% UI: 2.96–3.09] kg in June with troughs at 1.95 [95% UI: 1.88–2.02] kg in December (Fig. 2A and Supplementary Table 2). Consumption of vegetables exhibited seasonality as well, with the lowest intake in July and the highest in January (Fig. 2B). Compared to 2011–12, seasonal fluctuations have narrowed, suggesting improved supply chain efficiency and year-round availability.

**Fig. 3:**
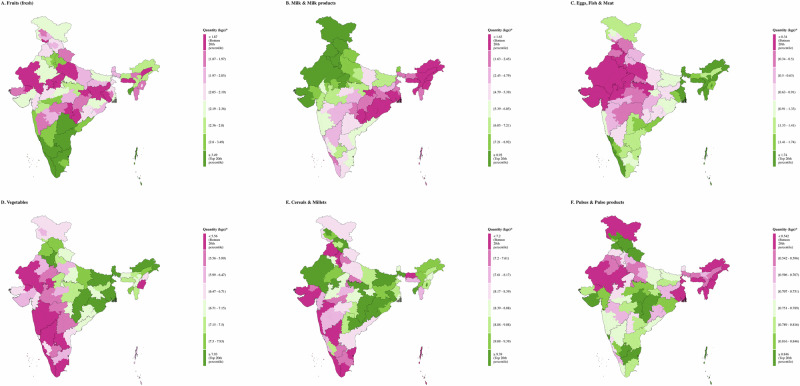
Inter-Regional Variations in Monthly Consumption (in terms of Adult Female Equivalent), 2023–24. **A** Fruits (fresh). **B** Milk and Milk Products. **C** Eggs, Fish and Meat. **D** Vegetables. **E** Cereals and Millets. **F** Pulses.

Geographic disparities in food consumption were evident in 2023–24 (Fig. 3). Fresh fruit intake was highest in southern states such as Kerala (Fig. 3A). At the same time, dairy consumption showed a north/west–south/east divide, with higher intake in northern and western regions and the lowest in the eastern and southern areas (Fig. 3B). Flesh product consumption was concentrated in the northeast, eastern coastal states, and Kerala (Fig. 3C). Vegetable intake was elevated in eastern states (Fig. 3D), cereals dominated in the east and north (Fig. 3E), and pulses were more prominent in central and southern regions (Fig. 3F).

**Fig. 4:**
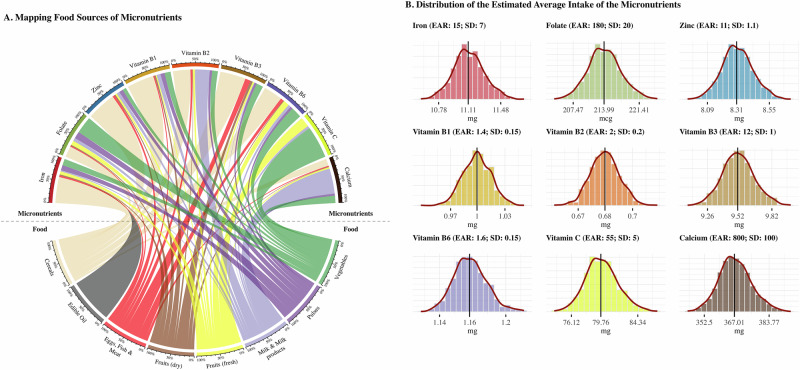
Mapping Dietary Sources of Micronutrients. **A** Mapping Food Sources of Micronutrients. **B** Distribution of the Estimated Average Intake of the Micronutrients.

Figure 4A shows the contribution of different food groups to selected micronutrients. Despite diversification, cereals remain the dominant source of several micronutrients, accounting for 40–60% of total intake for Iron, Folate, Zinc, and B vitamins (Fig. 4A). Pulses added 10–20% of Iron and Zinc, while fruits and vegetables contributed over 60% of Vitamin C. Dairy products accounted for 70–75% of Calcium. Animal-source foods, though rich in bioavailable Iron, are consumed infrequently by lower-income households. Pulses and vegetables contribute meaningfully to Iron and Folate intake, but their consumption levels remain insufficient to meet recommended dietary allowances.

We also estimate the distribution of the mean intake of micronutrients. For Iron, we found the mean intake to be 11.11, while the lower 2.5% bound was estimated at 10.78, and the upper 97.5% bound was estimated at 11.48. These results are reported in Fig. 4B. The normal distribution of iron requirement for adult women (non-lactating) has an Estimated Average Requirement (EAR) of 15 and a Standard Deviation (SD) of 7 based on the ICMR-NIN report on “Nutrition Requirements for Indians” [7]. We used the distribution of micronutrient requirements and estimates of micronutrient intake to compute the probability of inadequate micronutrient intake for non-lactating adult women. These results are reported in Supplementary Fig. 1. For Iron, we estimated the probability of inadequacy for a non-lactating adult female to be 69.2% (the 2.5% lower and the 97.5% upper bounds were 67.4% and 70.8%, respectively).

Moreover, we examined the dietary diversity of food items that contribute to the daily Iron intake per AFE across states in India. The results are reported in Supplementary Fig. 2. We found that the contribution of cereals to daily Iron intake varied significantly across the states. For example, in Rajasthan, where the estimated mean intake of Iron was 16.49 [95% UI: 16.34 to 16.62] mg per AFE, cereals contributed approximately 80% to the daily Iron intake. While in Nagaland, with an estimated mean Iron intake of 7.24 [95% UI: 7.03 to 7.49] mg per AFE, cereals contributed approximately 28% of the iron intake, while flesh products (eggs, fish & meat), and vegetables contributed more than 50% of the daily Iron intake.

## Discussion

India’s dietary landscape is undergoing a profound transformation, driven by rising incomes, shifting consumption patterns, and targeted welfare initiatives. The present analysis provides updated estimates of food group and micronutrient intakes in India, with a focus on disparities by place of residence, socioeconomic status, and season. The patterns, summarised in Tables 1 and 2, reveal both the magnitude and distributional shifts in consumption over the past decade. For example, the richest-to-poorest fruit intake ratio narrowed from 4.05 to 2.64, and seasonal gaps in several food groups also diminished, suggesting modest progress toward dietary equity.

The decline in food expenditure as a proportion of total household spending—falling below 50% in rural areas for the first time in post-independence India—signals improved living standards and economic maturation. This trend parallels patterns observed in other emerging economies undergoing nutrition transition, where staple-centric diets give way to more diverse and nutrient-rich consumption [22].

The sharp reduction in cereal expenditure, particularly among the bottom quintiles, reflects both demand-side shifts and the impact of large-scale food security programs such as the Pradhan Mantri Garib Kalyan Anna Yojana (PMGKAY) [23] and the National Food Security Act (NFSA) [24]. These schemes have effectively decoupled staple access from household budgets, enabling reallocation toward fruits, dairy, and animal-source foods. This reallocation is evident in the increased consumption of fresh fruits, milk, and meat across all socioeconomic strata, with the most pronounced gains among the poorest households. These shifts indicate the early success of targeted social protection measures and improved market integration in promoting more inclusive dietary improvements. Such patterns also align with the concept of “saved expenditure” acting as a fiscal stimulus for dietary diversification [25].

Existing studies have made substantial progress in documenting dietary patterns and nutrition outcomes in low– and middle–income countries [26, 27]. A systematic review by Mayén et al. (2014) [28], which included 33 studies from 17 LMIC countries, primarily from Brazil, China, and Iran, found that households with higher socioeconomic status consumed higher quantities of fruits and vegetables and had a more diversified diet. We found similar patterns in the context of India; however, the inequality in consumption of food items such as fresh fruits, dairy, and flesh products (eggs, fish & meat), between the top 20% and the bottom 20% has decreased significantly between 2011–12 and 2023–24. At the same time, food consumption in 2023–24 revealed pronounced regional disparities—fruit intake was highest in the south, dairy in the north and west, flesh foods in the northeast and Kerala, vegetables in the east, cereals in the north and east, and pulses in central and southern states. This heterogeneity reflects cultural preferences, agroecological conditions, and market access, highlighting the importance of regionally tailored nutrition strategies [29].

While the shift toward greater dietary diversity is encouraging, it remains insufficient to meet recommended intake levels for many essential nutrients. Significant micronutrient gaps persist across income groups and regions, particularly for Iron, Zinc, B–Vitamins (Vitamins B1, B2, B3, and B6), and Calcium. These findings highlight that diversification alone does not ensure nutritional adequacy; complementary strategies—such as targeted subsidies, focused interventions, and strengthened supply chains—are essential to translate evolving consumption patterns into meaningful health gains.

Encouragingly, the most considerable relative improvements in dietary diversity have occurred among the poorest quintiles, contributing to a narrowing of both rural–urban and socioeconomic gaps. National-level analyses (survey on Household Consumption Expenditure Survey 2023–24) indicate that overall rural–urban consumption disparities decreased from 84% to 70% over the past decade. Our study corroborates this convergence through food group–specific trends: the steepest proportional gains in fruit, dairy, and animal-source food intake were observed among the bottom quintiles.

Seasonal fluctuations in perishable food intake—particularly fruits and vegetables—have diminished significantly since 2011–12, suggesting improvements in cold chain infrastructure, storage, and transport logistics. These supply-side enhancements have enabled year-round access to nutrient-dense foods, even in remote regions. Notably, India’s horticultural output has surged in recent years, with the gross value added from fruits, vegetables, and floriculture surpassing that of cereals for the first time—reflecting both production growth and shifting consumption patterns [30]. The narrowing of seasonal troughs and peaks is a critical marker of food system resilience and has implications for dietary stability and micronutrient adequacy [31].

However, despite these gains, significant challenges remain in ensuring that perishable foods are consistently accessible and affordable across geographies and income groups. Gaps in last-mile connectivity, uneven market integration, and affordability constraints continue to limit the nutritional impact of these supply-side improvements.

Despite the persistence of cereals as dominant contributors to Iron, Zinc, and B Vitamins intake, their limitations in providing key micronutrients—especially vitamin C and Calcium—are evident. Micronutrient mapping based on HCES data and compositional analysis reveals that fruits and vegetables contribute over 60% of vitamin C. In comparison, dairy accounts for 70–75% of Calcium. Pulses and legumes, while modest in quantity, also play a critical role in Zinc and Folate intake, especially among vegetarian households.

However, the quantitative estimates of micronutrient intake highlight that the nutritional gaps persist despite modest improvements in dietary diversity. For example, our analysis shows that the mean iron intake among non-lactating adult women falls short of the EAR by nearly 4 mg/day, with almost 70% of this population group at risk of inadequate intake (Supplementary Fig. 1). Such high probabilities of inadequacy underscore the limitations of current dietary patterns and the need for targeted interventions that address both quantity and quality of nutrient intake. These findings also reinforce the importance of integrating nutrient requirement distributions into population-level dietary assessments, enabling more precise identification of at-risk groups and more effective policy responses.

Furthermore, we also observed that in many large states, the Iron intake of households primarily comes from cereals. For example, in Rajasthan, cereals contribute approximately 79% of the Iron intake, while in Uttar Pradesh, Haryana, Punjab, Bihar, Gujarat, Maharashtra, Madhya Pradesh, it is in excess of 60%. This raises the question of absorption of non–heme iron which is substantially lower (3-5%) compared to heme iron sources. The Comprehensive National Nutrition Survey *(CNNS, 2016–18)* further highlights that over 25–30% of adolescents are iron deficient, reinforcing that reliance on cereal-based iron intake alone is insufficient to meet physiological needs [32].

These findings underscore the significance of dietary diversity in mitigating micronutrient deficiencies and alleviating the double burden of malnutrition [8, 26, 33]. They also highlight the need for programmatic strategies that promote access to nutrient-dense foods beyond cereals, including fruits, vegetables, dairy, pulses, and animal-source foods—each of which contributes uniquely to the micronutrient landscape.

Interpretation of these findings must reflect the population-level nature of HCES-based analysis. Household acquisition data do not capture intra-household allocation or foods consumed outside the home, nor do they allow consideration of cooking losses — characteristics also highlighted in national dietary guidance. In addition, ultra-processed foods and out-of-home foods are not included in nutrient estimation due to lack of composition coding in HCES, even though their contribution to diets is rising. Dietary identity, including vegetarianism, which shapes animal-source food choices for cultural reasons rather than affordability alone, is not observed in HCES. Hence, trends in animal-source food intake should be interpreted as changes among households that consume them, not necessarily all households.

Taken together, these considerations highlight the role of HCES as a complementary surveillance platform. While 24-h recalls and biomarker assessments remain essential for measuring individual adequacy and deficiency, HCES uniquely enables regular, nationally representative monitoring of dietary sourcing and inequities at a scale not feasible through individual-level nutrition surveys. Policies designed to strengthen nutrition security should therefore be informed by combined evidence from food system surveillance (HCES) and individual nutrition assessment tools.

### Policy implications and future directions

The dietary intake findings underscore the need for a multi-pronged policy response that reflects both evolving consumption patterns and persistent nutritional gaps. First, policies should be implemented to actively support the production, distribution, and affordability of nutrient-rich foods, including fruits, vegetables, pulses, dairy products, and animal-source products [34]. Second, nutrition guidelines should be regionally adapted to account for cultural preferences, agroecological diversity, and observed consumption heterogeneity—particularly in states with lagging intake of key food groups. Third, targeted subsidies and market incentives for perishable, micronutrient-dense foods could enhance access among vulnerable populations, building on the existing equity gains. Fourth, continued investment in cold chain infrastructure and transport logistics is essential to sustain year-round availability and reduce seasonal volatility in intake [35].

At the same time, nutritional surveillance and reformulation standards of processed and packaged foods—whose consumption is rising rapidly across all quintiles—must ensure nutritional quality and mitigate risks associated with ultra-processed diets [36]. Nutrition coding updates would enable surveillance systems to better capture their impact on micronutrient adequacy and unhealthy dietary risks. Future research should investigate the health implications of shifting food environments, the role of diverse food systems in preventing non-communicable diseases, and the effectiveness of integrated, regionally tailored interventions in sustaining equitable nutrition gains. Periodic 24-h recalls, and biomarker surveys are essential for assessing adequacy and deficiency. Complementing these with HCES would provide a stronger evidence base for nutrition. Equally critical is the regular generation of more granular dietary data—disaggregated by state, region, physiological group, and gender—to inform context-sensitive strategies and program targeting that address persistent gaps in nutrient adequacy. Together, these policy directions offer a roadmap for advancing nutrition equity and resilience in India’s evolving food system.

### Limitations

The use of HCES for dietary surveillance and program design offers several advantages. The large sample size and regular implementation allow for monitoring trends over time and across subnational units. It also enables the linking of dietary patterns with socioeconomic and demographic variables for more nuanced targeting of interventions. Integrating HCES-derived nutrient intake estimates can help prioritise investments in fortification, supplementation, and food system interventions that address both undernutrition and emerging diet-related non-communicable diseases.

Despite these benefits, several limitations warrant consideration. First, the estimates of the quantity of food consumed are based on household surveys, where the unit of observation is the household and not individual members; therefore, intra-household distributions are not captured. This is particularly relevant for nutrients like Iron, where standardising intake to AFE may overestimate the prevalence of inadequacy [2, 37]. Second, the survey is based on 7-day and 30-day recall, and hence, is subject to bias if items are not recalled accurately. The analysis assumes that foods obtained during the recall period are consumed within that same interval. However, the data do not allow us to observe household storage, wastage, or sharing. Third, micronutrient estimates are derived from the nutrient composition of raw foods from the IFCT 2017. There is a possibility that nutrient consumption based on raw food consumption may be inaccurate since many Indian cooking practices may lead to significant losses in nutrient content of food. Furthermore, our analysis does not include the nutritional content of food consumed outside the house, beverages, served processed food, or packaged processed food because standardised food composition for these products are not available in HCES. Fourth, the HCES does not capture dietary identity, including vegetarianism, which strongly shapes consumption of animal-source foods in India. In addition, the surveys under-represent households at the very top of the income distribution, likely leading to an underestimation of inequality among the richest groups. Finally, these findings should be interpreted as population-level indicators of dietary patterns and potential micronutrient adequacy. It is also important to mention that for robust estimation of micronutrient intake, one has to account for the day-to-day variability. Typically, to capture this, one has to collect the nutrition intake information over several days. Moreover, such diet and nutrition surveys depend on 24-h dietary recall at the level of the individual; if this is not possible, then perhaps use the food diary record [7]. Therefore, our results suggest broad patterns in dietary sourcing and its associated micronutrient intake, but not precise measures of nutritional status. For a more precise and rigorous estimation, a detailed nutrition survey representative of the states and the regions within large states would be required.

## Supplementary information

Supplementary

## Data Availability

Data from HCES 2011-12 and 2023-24 are available via the NSSO microdata catalogue [20].
