# Supplementary material for: Food consumption patterns and their micronutrient content in India: Evidence from the household consumption expenditure surveys, 2011–12 and 2023–24
Source: Eur J Clin Nutr. 2026 Apr 7;80(6):583–93. doi: 10.1038/s41430-026-01732-3 (PMC13286997; doi:10.1038/s41430-026-01732-3)
Supplement: Supplementary file 1 — Supplementary [file 41430_2026_1732_MOESM1_ESM.docx]

# Supplementary

**Section 1: Construction of Adult Female Equivalent**

We use household demographics data (gender, age, and presence of children under one year) to construct the household size in terms of the adult female equivalent, based on energy requirements. For example, a household with a 30-year-old male and a 30-year-old female has an energy requirement of 2,710 for the moderately active male, while the adult female's requirement is 2,130. Then, the adult male is approximately 1.3 times the size of an adult female. In terms of the adult female equivalent, the household size is 2.3. The energy requirements are based on the ICMR-NIN 2020 report, "Nutrition Requirements for Indians" (see the table below).

**Supplementary Table 1: Energy Requirements**

| **Energy Requirement**  **(Kcal/day)** | | **Assumption** |
| --- | --- | --- |
| Child 0 to 12 months (= 0 years) | 595 | 0-6mo 530 & 6-12mo is 660: we  took the average |
| Child 1 to 3 yrs | 1110 |  |
| Child 4 to 6 yrs | 1360 |  |
| Child 7 to 9 yrs | 1700 |  |
| Girls 10 to 12 yrs | 2060 |  |
| Boys 10 to 12 yrs | 2220 |  |
| Girls 13 to 15 yrs | 2400 |  |
| Boys 13 to 15 yrs | 2860 |  |
| Girls 16 to 18 yrs | 2500 |  |
| Boys 16 to 18 yrs | 3320 |  |

| Adult female | 2130 | Moderate Work |
| --- | --- | --- |
| Adult female (lactating) | 2690 | If a child (age = 0 years) is present in the household, then women aged between 19 and 49 years are assigned this value, depending on the number of children in the household. We took the average of the additional 600 for lactating women with a child 0 to 6 months & 520 for (6 to 12 months) = 2130  + 560 = 2690 |
| Adult men | 2710 | Moderate Work |

**Section 2: Estimating the seasonal variation in consumption intake of Fresh Fruits and**

**Vegetables**

**Supplementary Table 2: Seasonality in consumption (in terms of AFE) 2011-12 & 2023-24**

| Food Group | Month | Quantity (kg per AFE) consumed in a month | | | |
| --- | --- | --- | --- | --- | --- |
|  |  | **Rural** | | **Urban** | |
|  |  | **2011–12** | **2023–24** | **2011–12** | **2023–24** |
| Fresh Fruits | **January** | 1.31 (1.19–1.43) | 1.97 (1.9–2.03) | 2.27 (2.06–2.5) | 2.94 (2.82–3.07) |
|  | **February** | 1.46 (1.33–1.61) | 1.96 (1.89–2.02) | 2.28 (2.04–2.53) | 3.07 (2.91–3.22) |
|  | **March** | 1.45 (1.33–1.6) | 2.26 (2.19–2.34) | 2.66 (2.35–2.96) | 3.39 (3.26–3.54) |
|  | **April** | 1.2 (1.07–1.33) | 2.73 (2.65–2.82) | 2.43 (2.21–2.68) | 4.13 (3.97–4.31) |
|  | **May** | 1.82 (1.7–1.93) | 2.85 (2.78–2.92) | 2.87 (2.68–3.08) | 4.15 (4–4.31) |
|  | **June** | 2.32 (2.15–2.49) | 3.02 (2.96–3.09) | 2.97 (2.71–3.23) | 3.85 (3.72–3.98) |
|  | **July** | 2.52 (2.36–2.71) | 2.62 (2.54–2.7) | 3.05 (2.82–3.29) | 3.26 (3.1–3.41) |
|  | **August** | 2.16 (2.02–2.3) | 2.21 (2.09–2.33) | 2.9 (2.72–3.11) | 3.25 (2.99–3.55) |
|  | **September** | 1.63 (1.51–1.76) | 2.03 (1.91–2.14) | 2.67 (2.45–2.92) | 2.99 (2.75–3.26) |
|  | **October** | 1.69 (1.56–1.85) | 2.14 (2.07–2.23) | 2.51 (2.31–2.72) | 3.02 (2.87–3.18) |
|  | **November** | 1.5 (1.39–1.62) | 2.15 (2.08–2.23) | 2.44 (2.25–2.64) | 2.98 (2.84–3.13) |
|  | **December** | 1.35 (1.22–1.49) | 1.95 (1.88–2.02) | 2.41 (2.19–2.66) | 2.88 (2.75–3.02) |
|  |  |  |  |  |  |
| Vegetables | **January** | 7.24 (7.02–7.45) | 7.44 (7.34–7.55) | 7.67 (7.39–7.9) | 7.34 (7.16–7.5) |
|  | **February** | 7.33 (7.16–7.53) | 7.3 (7.2–7.4) | 7.43 (7.17–7.67) | 7.29 (7.11–7.46) |
|  | **March** | 7.14 (6.95–7.32) | 6.97 (6.88–7.07) | 6.48 (6.17–6.77) | 6.78 (6.63–6.92) |
|  | **April** | 6.57 (6.38–6.76) | 6.72 (6.63–6.8) | 6.48 (6.26–6.7) | 6.8 (6.68–6.92) |
|  | **May** | 6.5 (6.36–6.65) | 6.68 (6.59–6.76) | 6.84 (6.65–7.05) | 6.77 (6.64–6.89) |
|  | **June** | 6.74 (6.56–6.92) | 6.58 (6.5–6.67) | 6.78 (6.52–7.05) | 6.5 (6.36–6.62) |
|  | **July** | 5.87 (5.7–6.06) | 6.36 (6.27–6.46) | 5.8 (5.61–6) | 6.2 (6.05–6.34) |
|  | **August** | 5.59 (5.44–5.76) | 6.61 (6.45–6.76) | 6.18 (5.99–6.38) | 6.55 (6.36–6.76) |
|  | **September** | 5.81 (5.66–5.94) | 6.6 (6.44–6.75) | 6.23 (6.02–6.47) | 6.41 (6.21–6.6) |
|  | **October** | 6.2 (6.03–6.38) | 6.61 (6.5–6.72) | 5.91 (5.74–6.1) | 6.54 (6.4–6.67) |
|  | **November** | 6.23 (6.05–6.4) | 6.68 (6.58–6.77) | 6.55 (6.36–6.75) | 6.59 (6.46–6.72) |
|  | **December** | 7.02 (6.84–7.22) | 7.07 (6.97–7.18) | 7.12 (6.86–7.37) | 6.89 (6.76–7.03) |
| Note: 95% Uncertainty intervals are reported in parenthesis. | | | | | |

# Section 3: Conversion of Food Items into Micronutrients

To convert the food items into their micronutrient values, we used the information on the micronutrients for the food item from the ICMR–National Institute of Nutrition (ICMR–NIN) report on Indian Food Composition Tables (2017) (28). The Indian Food Composition Tables (2017) provide micronutrient values per 100 grams of each sub-item in the broad food category. For instance, 100 grams of potatoes provide 0.55 mg of Iron. We combined this micronutrient information with the actual consumption of potatoes to calculate the iron intake from potatoes.

It is important to note that the quantity of consumption of bananas, dry coconut, green coconut, orange, lemon, egg, and milk was not recorded in kilograms in the dataset. To address this, we utilized average weights for these items: banana (1 unit = 118 g), dry coconut (1 unit = 980g), green coconut (1 unit = 1300 g), orange (1 unit = 154 g), lemon (1 unit = 65 g), egg (1 unit = 44 g), and milk (1 liter of light milk = 1032 g).

# Section 4: Estimating the Prevalence of Inadequacy of the Micronutrient Intake

The daily micronutrient intake associated with food items was modelled similarly to the intake of food items, and the analysis was in terms of the AFE. For each micronutrient, we used the Estimated Average Requirement (EAR) for Adult females (non-lactating) and the Recommended Dietary Allowances (RDA) (which is equal to EAR + 1.96*Standard Deviation) and constructed the normal distribution of the requirements of the micronutrient; however, for Iron, we took the log-normal distribution (29). Using the distribution and estimates of micronutrient intake (in terms of AFE), we estimated the probability of inadequacy for each micronutrient intake. The table below reports the EAR and the RDA from the ICMR-NIN 2020 report on "Nutrition Requirements for Indians" (7). The report provides a detailed discussion on how the EAR and RDAs were estimated.

**Supplementary Table 3: EAR and RDA for moderately working adult females (non–lactating)**

| **Micronutrient** | **EAR** | **RDA** |
| --- | --- | --- |
|  |  |  |
| Iron | 15 | 29 |
| Folate (Vitamin B9) | 180 | 220 |
| Zinc | 11 | 13.2 |
| Vitamin B1 | 1.4 | 1.7 |
| Vitamin B2 | 2 | 2.4 |
| Vitamin B3 | 12 | 14 |
| Vitamin B6 | 1.6 | 1.9 |
| Vitamin C | 55 | 65 |
| Calcium | 800 | 1000 |

**Supplementary Figure 1: Probability of Inadequacy (in terms of Adult Female Equivalent)**

**
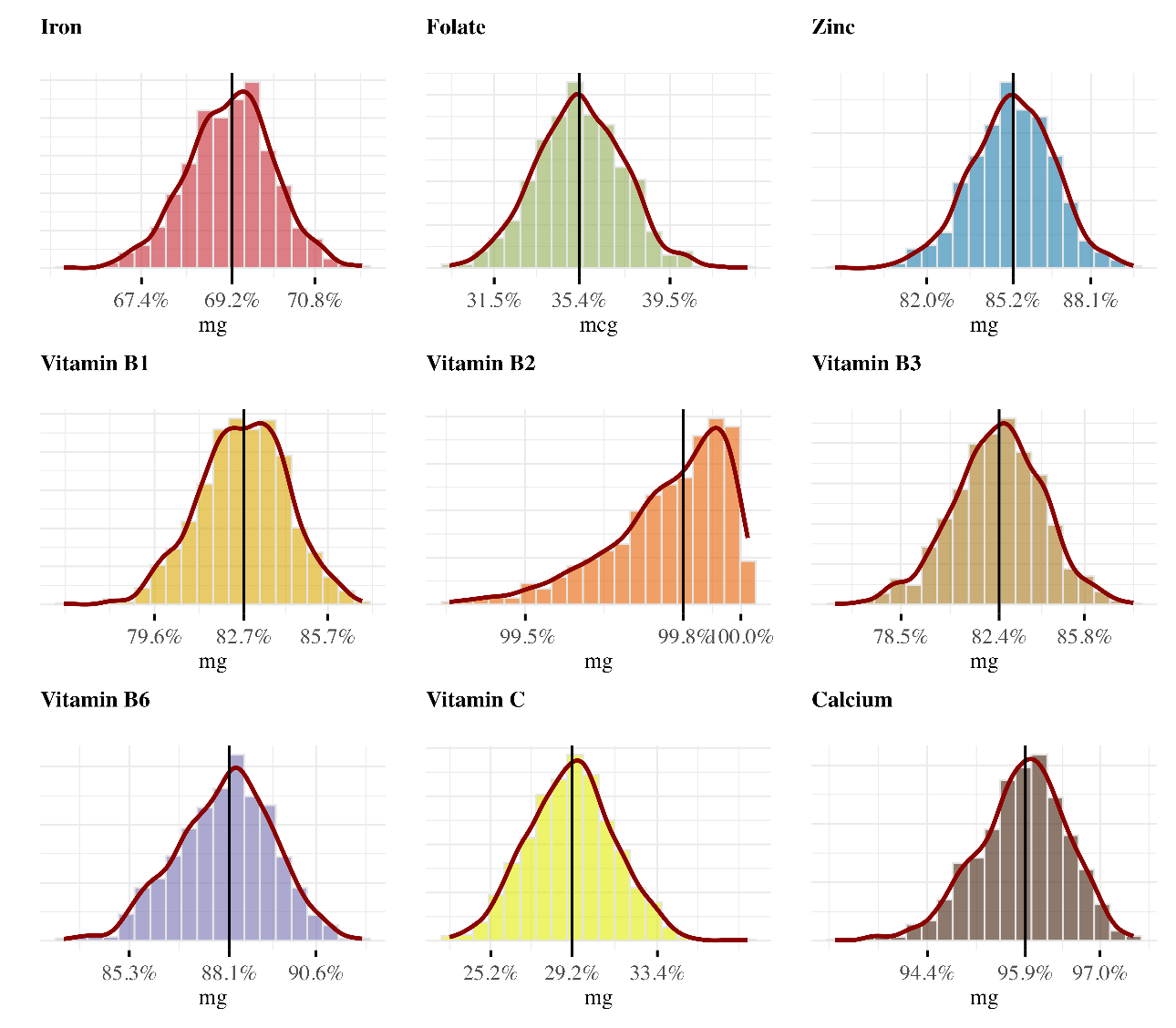
**

**Note:** The number in the x-axis associated with the black line is the median value, and the number on the left and right of the median value reflects the 2.5% and the 97.5% values, respectively, of the estimated distribution of the intake.

*For Iron, we used the log-normal distribution of requirement, while for other micronutrients, we used the normal distribution of requirement.

**Supplementary Figure 2:**

| 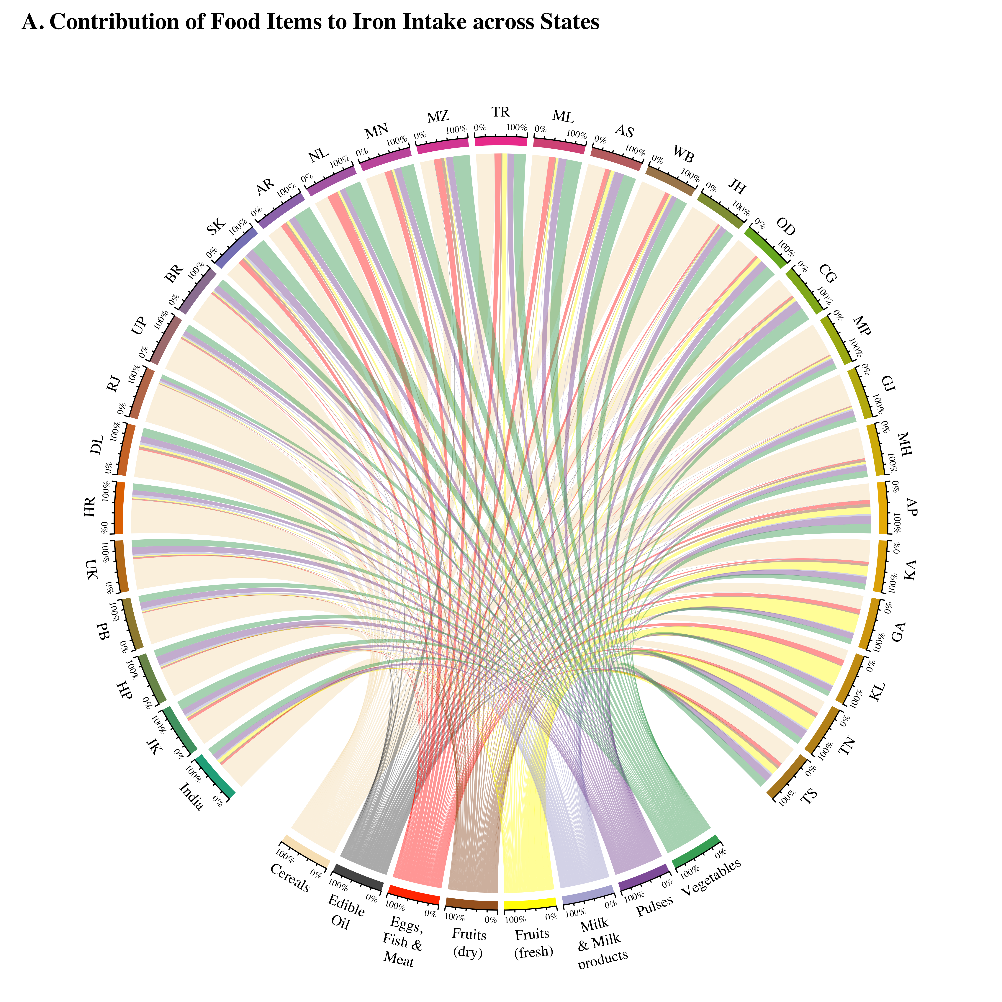 | | | | | | | | |
| --- | --- | --- | --- | --- | --- | --- | --- | --- |
| **B. Estimated Daily Mean Intake of Iron with 95% Uncertainty Range across States and Contribution**  **of Food Items to Iron Intake** | | | | | | | | |
|  | | | | **Contribution of Food Items to the Iron Intake of the Household** | | | | |
|  |  | **Estimated Daily Mean Intake*** | **95% Uncertainty Interval** | **Cereals** | **Eggs,**  **Fish & Meat** | **Fruits (fresh)** | **Pulses &**  **Pulse products** | **Vegetables** |
|  | | | |  | | | | |
|  | India: India | 11.11 | (11.07 to 11.16) | 58.2% | 3.4% | 6.9% | 11.6% | 14.6% |
|  | JK: Jammu and Kashmir | 10.62 | (10.25 to 10.98) | 55.6% | 4.8% | 2.1% | 11.8% | 18.4% |
|  | HP: Himachal Pradesh | 14.01 | (13.74 to 14.29) | 59.7% | 1.6% | 1.6% | 17.1% | 14.1% |
|  | PB: Punjab | 13.16 | (13.03 to 13.28) | 64.4% | 1.1% | 2% | 12.3% | 14% |
|  | UK: Uttarakhand | 11.87 | (11.72 to 12.04) | 61.1% | 2% | 1.9% | 14.7% | 14.9% |
|  | HR: Haryana | 14.18 | (13.93 to 14.41) | 67.2% | 0.9% | 2.6% | 9.1% | 13.3% |
|  | DL: Delhi | 10.93 | (10.76 to 11.11) | 55.4% | 2.6% | 4% | 12.6% | 16.8% |
|  | RJ: Rajasthan | 16.49 | (16.34 to 16.62) | 79.5% | 0.5% | 1.9% | 5.2% | 8% |
|  | UP: Uttar Pradesh | 11.88 | (11.84 to 11.93) | 67.3% | 1.9% | 2% | 9.8% | 14.2% |
|  | BR: Bihar | 12.60 | (12.5 to 12.69) | 62.9% | 2.9% | 2.3% | 12.2% | 15.4% |
|  | SK: Sikkim | 9.00 | (8.57 to 9.42) | 32.2% | 9.6% | 2.8% | 17.3% | 29.1% |
|  | AR: Arunachal Pradesh | 7.81 | (7.57 to 8.07) | 31.1% | 10.7% | 5.4% | 13.5% | 35.3% |

|  | NL: Nagaland | 7.24 | (7.03 to 7.49) | 27.9% | 21.1% | 4.7% | 11.3% | 32.9% |
| --- | --- | --- | --- | --- | --- | --- | --- | --- |
|  | MN: Manipur | 5.98 | (5.84 to 6.13) | 39.2% | 12.6% | 4% | 13.4% | 26.9% |
|  | MZ: Mizoram | 6.56 | (6.32 to 6.82) | 27.6% | 14.8% | 2.8% | 13% | 34% |
|  | TR: Tripura | 7.94 | (7.78 to 8.1) | 36.8% | 12.5% | 9.3% | 13% | 23.5% |
|  | ML: Meghalaya | 6.21 | (6.06 to 6.38) | 33.4% | 12.4% | 4.3% | 15.5% | 31.1% |
|  | AS: Assam | 6.84 | (6.75 to 6.94) | 35.7% | 8.8% | 7.1% | 18.3% | 25.6% |
|  | WB: West Bengal | 9.31 | (9.23 to 9.38) | 51.5% | 8.1% | 4.6% | 10.8% | 22.3% |
|  | JH: Jharkhand | 9.35 | (9.2 to 9.49) | 61.1% | 3.5% | 1.8% | 13% | 17.6% |
|  | OD: Odisha | 8.75 | (8.61 to 8.89) | 46.9% | 4.3% | 9% | 15.5% | 21.6% |
|  | CG: Chhattisgarh | 8.39 | (8.22 to 8.56) | 41.3% | 3% | 6.5% | 18.1% | 26.5% |
|  | MP: Madhya Pradesh | 13.32 | (13.2 to 13.45) | 68.7% | 1.1% | 4.4% | 9.9% | 11.7% |
|  | GJ: Gujarat | 11.67 | (11.58 to 11.77) | 66.6% | 0.8% | 3.6% | 11.8% | 11.9% |
|  | MH: Maharashtra | 11.11 | (11.02 to 11.2) | 61.9% | 2.6% | 4.5% | 11.3% | 12.7% |
|  | AP: Andhra Pradesh | 7.79 | (7.7 to 7.87) | 32.8% | 7.6% | 13.7% | 16.6% | 18.5% |
|  | KA: Karnataka | 10.75 | (10.63 to 10.87) | 44.1% | 4.3% | 19.1% | 13.2% | 11.8% |
|  | GA: Goa | 14.41 | (13.92 to 14.94) | 27.4% | 8.6% | 37.1% | 11.9% | 10.6% |
|  | KL: Kerala | 10.67 | (10.53 to 10.82) | 21.2% | 11.5% | 41.6% | 11.6% | 10.1% |
|  | TN: Tamil Nadu | 8.58 | (8.48 to 8.67) | 26.2% | 6.5% | 28.4% | 17.9% | 13.8% |
|  | TS: Telangana | 8.16 | (8.04 to 8.28) | 37.5% | 7.6% | 14.4% | 15.3% | 16% |
|  | | | |  | | | | |

*Note: The estimated daily mean intake of Iron is standardized for Adult Female Equivalent, and the units are mg.
